# Resistance to Biocides in *Listeria monocytogenes* Collected in Meat-Processing Environments

**DOI:** 10.3389/fmicb.2016.01627

**Published:** 2016-10-19

**Authors:** Daniele Conficoni, Carmen Losasso, Enzo Cortini, Andrea Di Cesare, Veronica Cibin, Valerio Giaccone, Gianluca Corno, Antonia Ricci

**Affiliations:** ^1^Dipartimento per la Sicurezza Alimentare, Istituto Zooprofilattico Sperimentale delle VeneziePadova, Italy; ^2^Department of Animal Medicine, Production and Health, University of PaduaPadova, Italy; ^3^Microbial Ecology Group, National Research Council – Institute of Ecosystem Study (CNR-ISE)Verbania, Italy

**Keywords:** *Listeria monocytogenes*, food-processing plants, environment, resistance genes, gene expression, efflux pumps

## Abstract

The emergence of microorganisms exerting resistance to biocides is a challenge to meat-processing environments. Bacteria can be intrinsically resistant to biocides but resistance can also be acquired by adaptation to their sub-lethal concentrations. Moreover, the presence of biocide resistance determinants, which is closely linked to antibiotic resistance determinants, could lead to co-selection during disinfection practices along the food chain, and select cross-resistant foodborne pathogens. The purpose of this work was to test the resistance of wild strains of *Listeria monocytogenes*, isolated from pork meat processing plants, toward benzalkonium chloride (BC), used as proxy of quaternary ammonium compounds. Furthermore, the expression of two non-specific efflux pumps genes (*lde* and *mdrL*) under biocide exposure was evaluated. *L. monocytogenes* were isolated from five processing plants located in the Veneto region (northeast of Italy) before and after cleaning and disinfection (C&D) procedures. A total of 45 strains were collected: 36 strains before and nine after the C&D procedures. Collected strains were typed according to MLST and ERIC profiles. Strains sampled in the same site, isolated before, and after the C&D procedures and displaying the same MLST and ERIC profiles were tested for their sensitivity to different concentrations of BC, in a time course assay. The expression of non-specific efflux pumps was evaluated at each time point by qPCR using *tufA* gene as housekeeping. A differential expression of the two investigated genes was observed: *lde* was found to be more expressed by the strains isolated before C&D procedures while its expression was dose-dependent in the case of the post C&D procedures strain. On the contrary, the expression of *mdrL* was inhibited under low biocidal stress (10 ppm BC) and enhanced in the presence of high stress (100 ppm BC). These findings suggests a possible role for C&D procedures to select *L. monocytogenes* persisters, pointing out the importance of dealing with the identification of risk factors in food plants sanification procedures that might select more tolerant strains.

## Introduction

*Listeria monocytogenes* is an ubiquitous Gram-positive foodborne pathogen which causes listeriosis, a high pathogenicity clinical condition which occurs mainly among at-risk groups such as elderly people, pregnant women, immunocompromised people, and newborns (the so called YOPIs, young, old, pregnant, immunocompromised) (Maertens de Noordhout et al., 2014).

According to Havelaar et al. (2012), the burden disease of perinatal *L. monocytogenes* on an individual basis (Disability-Adjusted Life-Year—DALYs—per 1000 cases) is the highest burden among foodborne diseases in the Netherlands. The large meta analysis of Maertens de Noordhout and co-authors confirmed this trend, pointing out the high DALYs of *L. monocytogenes* at a global level (Maertens de Noordhout et al., 2014).

*Listeria* is able to survive and grow at low temperatures (0–4°C) even in food with high salinity and low water activity. This ability to persist and multiply in the food environment makes *Listeria* hard to control in plants producing food (Belluco et al., 2016).

According to Reg. 852/2004 (EU Commission, 2004), *the Hazard Analysis and Critical Control Point (HACCP) is an instrument to help food business operators attain a higher standard of food safety*. In this light, the cleaning and disinfection (C&D) procedures are a fundamental critical control point for the reduction of microbiological load in the production environment. Surface sanitization is undertaken with the aim of removing microorganisms and materials causing microbial growth (dirty and soiled surfaces), thus reducing the spoilage organisms, even extending the shelf life of some products. In the modern food industry, the high volume of food production together with the consumer demands for healthy, minimally-processed, nutritious, and foods devoid of additives, have had a high impact on the need of biocide use in food-processing plants (Condell et al., 2012).

Biocides (antiseptics, disinfectants, and preservatives) are regularly used at a domestic level and in the food industry with the aim of preventing bacterial contamination during food processing, to disinfect, sanitize, sterilize, and preserve materials or critical steps from microbial contamination and proliferation (Mavri et al., 2012). However, biocides must be managed correctly to avoid any loss of activity mediated by resistances and tolerances (SCENIHR, 2009).

In contrast with antibiotics, the exposure time to biocides is a matter of minutes and for some molecules the longer the time, the higher the killing rate. For this reason respecting the contact time indicated by manufacturers is of paramount importance. Incorrect exposure to disinfectants could lead to an incomplete eradication of bacterial contamination, with the consequent risk of bacterial re–colonization after the C&D procedures. The effectiveness of disinfectants is largely governed by the temperature used, which could vary greatly according to the facility and operator skills (Cerf et al., 2010). The organic matter load is another cause of disinfection failure, due to the inactivation of the disinfectant by the interfering organic matter (Cerf et al., 2010). Biocide resistance can be mediated by intrinsic or acquired mechanisms: intrinsic resistance is an innate bacteria characteristic coded by bacterial genome (species-specific) and includes membrane permeability, drug efflux pumps, biofilm formation and chemical transformation of toxic compounds (Nikaido, 2003; Poole, 2007; Pu et al., 2016). These strategies are also involved in the resistance against antibiotics (Thorrold et al., 2007).

The present work investigated the impact of C&D procedures actually performed in five meat-processing plants on *L. monocytogenes* persistence in the environments. The resistance of wild strains toward benzalkonium chloride (BC), used as proxy of quaternary ammonium compounds was assessed. Moreover, the expression of the two non-specific efflux pumps genes *lde* (*Listeria* drug efflux) and *mdrL* (Multi-Drug Resistance *Listeria*) under biocide exposure was evaluated.

## Materials and methods

### Identification of pork meat processing plants

Five pork meat-processing plants were selected with the assistance of the Local Health Authority. The plants were selected in order to account for the whole range of plants production size of the Veneto region of Italy (northeast of the Country) being representative of small, lower intermediate, intermediate, upper intermediate and large production plants (1 plant for each size category, according to expert opinion). The dimension of the processing space was used as proxy of the size of the production plants.

### Hygiene assessment questionnaire

Hazard Analysis and Critical Control Points (HACCP) manuals were evaluated for their coherence with International Life Sciences Institute Research guidelines (ILSI, 2005). A checklist for each plant was created, taking into consideration critical procedures. The customized checklist was used to assess the cleaning and disinfection procedures (C&D) performed after each production cycle. The entire checklists are available under request.

### Sampling procedures

Each selected plant was sampled before and after the C&D procedures. Sampling was performed to assess the presence of *L. monocytogenes* before and after C&D procedures. To fulfill this purpose, the following surfaces/environments were sampled: broom, the containers for processed raw meat, the water-drainage border surface, the corner between the wall, and the floor of the processing room, the drying space, the floor, the moving hooks, the kart containing meat, the meat kneader, the knives, the drainage wells of the refrigerating, and processing rooms, the needle meat aerator, the meat mincer, the pallet containing bacon, the refrigerating room, the rope for hook mobility, the salami tying, the sausage maker, the sharpening steel, the sinks, spatulas, tables (upper and lower surfaces), the processing room walls, and the warehouse. The sampling was performed using *Whirlbag* sponge, rehydrated before use with 9 ml of an acqueous solution of Sodium Chloride (0.9%) and kept at refrigeration temperature (4°C) until analysis. All the samples were processed within 24 h from the sampling.

### Isolation and identification of *Listeria monocytogenes*

The isolation and characterization of *L. monocytogenes* were carried out according to EN ISO 16140 and UNI EN ISO 11290-1:2005. Briefly, the sponges were resuspended in 100 ml of Half Fraser Broth (HFB) and incubated at 30°C. After 24 h, 1.5 ml of each sample was collected and used for *L. monocytogenes* detection using the Bio-Rad IQ-Check- *L. monocytogenes* II kit (Biorad, USA). Positive samples were plated on ALOA agar and on Oxford agar media. A number of five colonies with the typical *L. monocytogenes* appearance were tested for haemolysis on Blood agar medium and finally tested by Christie Atkins Munch-Petersen test (CAMP test). Positive colonies were confirmed by Bio-Rad IQ-Check- *L. monocytogenes* II kit (Biorad, USA). Confirmed colonies were stored at −80°C in Microbank^TM^ (Pro-Lab Diagnostics, US).

### Antibiotics susceptibility testing

A subsample of *L. monocytogenes* strains (one colony for each tested surface) was tested for antibiotic susceptibility by using a commercial microdilution test (Sensititre® Streptococcus panel ITST4F) against a panel of 15 antimicrobials: ampicillin, cefotaxime, ceftriaxone, clindamicin, daptomicin, doxiciclin, eritromicin, levofloxacin, linezolid, meropenem, moxifloxacin, penicillin, teicoplanin, trimetoprim/sulfametoxoazol, and vancomycin, according to the manufacturer's recommendations. Results were assessed after 48 h of incubation at 37°C. The minimum inhibitory concentration (MIC) was defined as the lowest concentration of the antimicrobial that completely inhibited visible growth. The results were thus analyzed according to the cut-offs set by EUCAST (www.eucast.org).

### Genotypic assay

Bacterial DNA extracted from the selected collection of strains was examined for the presence of the following panel of genes of resistance to biocide and heavy metals: *cadA1-Tn5422* (Mullapudi et al., 2010); *cadA2-pLM80* (Ratani et al., 2012); *cadA3-EGDe* (Ratani et al., 2012); *LMOSA_2330*; *LMOSA_2220*; *pLI37*; *F2365_2257*; *mdrL, lde*. The primer sequences are listed in Table S1.

### Typing of the *Listeria monocytogenes* isolates

*Listeria monocytogenes* isolates were typed by means of Multi Locus Sequence Type (MLST) (Salcedo et al., 2003) and enterobacterial repetitive intergenic consensus (ERIC). MLST was performed according to the Pasteur Institute guidance (http://bigsdb.web.pasteur.fr/listeria/primers_used.html). PCR- amplicons were visualized on agar gel electrophoresis and sequenced for allele identification. The MLST profile was then identified using the Pasteur Institute database.

For ERIC-PCR typing, 2.5 μl of DNA template was added to a reaction mix containing 5 μl of Green Buffer (Promega), 0.3 mM each PCR Nucleotide Mix 1 μM of each primer and 0.3 μl of GoTaq; the reaction volume was made up to 25 μl with autoclaved and filtered Milli Q water (Millipore, Billerica, MA, USA). PCRs were carried out using a My Cylcer thermocycler (Bio- Rad). Primer sequences ERIC- 1R (ATGTAAGCTCCGGGGATTCAC) and ERIC2 (AAGTAAGTGACTGGGGTGAGCG) were designed by Versalovic et al. (1991). The ERIC- PCR profiles were obtained by electrophoresis of the different amplicons for 6 h at 60 V, in 2% agarose Tris borate- EDTA (TBE) gel stained with Midori Green (Bulldog Bio). A 100 bp DNA ladder H3 RTU (Nippon Genetics, Germany) was used as the PCR fragment size marker.

### Gene expression profiling under biocidal stress

The gene expression of two different strains collected in the same plant before and after the cleaning and disinfectant procedures was assessed, which belonged to the same MLST group and with the same genotyping profile. The strains were incubated at 37°C in the presence of 0, 10, and 100 ppm of Benzalkonium Chloride in Mueller Hinton Broth (MHB). The expression of two broad specificity drug efflux pumps, *mdrL* and *lde*, that confer resistance to a wide spectrum of biocides (Ortega Morente et al., 2013), and of *tufA* as housekeeping gene, was evaluated in a time course experiment after 0, 1, 4, and 24 h of incubation. At every time point, the total RNA was extracted by a QIAGEN RNAeasy kit plus RNA Protect Bacteria as suggested by the manufacturer (QIAGEN). Extracted RNA was quantified by Nanodrop, and then immediately retrotranscripted using the QuantiTect-Reverse Transcription (QIAGEN). The obtained cDNA was stored until use at −20°C. The qPCRs were performed according to Di Cesare et al. (2015), except for the expression of results. The Non Reverse Transcripted sample (NoRT) was analyzed along with the retrotranscripted samples. Test specificity was tested by means of melting profile and gel electrophoresis. The adjusted Ct (aCt) was calculated as follows:

$$\begin{array}{l} \text{aCt }=\;{\text{log}}_{2}\left(2^{\text{Ct}}\;-\;2^{\text{NoRTCt}}\right) \end{array}$$

where Ct was the cycle threshold of the retrotranscripted sample and NoRTCt was the cycle threshold of the Non Reverse Transcripted sample. The resulted value was used for the data analysis. Each experiment was repeated twice.

### Data analysis

The adjusted resulted Cts were averaged and used to obtain a ΔΔCt value according to Livak and Schmittgen (2001).

## Results

### Hygiene assessment questionnaire

The hygiene assessment highlighted a series of critical action disregarded during C&D procedures. As it can be noticed from Table 1, the actions neglected by three or more processing plants were: the correct use of disinfectant concentration, the correct exposure time to cleaning agents, the control of rinsing water temperature, the appropriate use of cleaning nozzles, avoidance of aerosol formation, changing of work clothes between cleaning, and disinfection procedures, avoidance of raw meat debris persistence, the suitability of the dimension of drainage holes for debris, all the surfaces of the plants cleaned and drainage wells used appropriately. Interestingly four of the five investigated plants did not use the correct biocide concentration during C&D procedures, nor avoided the persistence of raw meat debris.

**Table 1:** **Critical action disregarded during C&D procedures**.

	**Plant 1**	**Plant 2**	**Plant 3**	**Plant 4**	**Plant 5**
Use of products reported in the manual	no	Yes	Yes	no	yes
Disinfectant type	Sodium hydroxide and sodium hypochlorite	Sodium peroxide, hydrogen peroxide, peracetic acid, acetic acid	Sodium hypochlorite	Didecyl-Dimethyl Ammonium Chloride	Peracetic acid, hydrogen peroxide, acetic acid
Disinfectant dose	1%	0.5–1%	0.1%	4%	2%
Disinfectant exposure time	5–10 min	until the next processing	1 min	until the next processing	10 min
Disinfectant concentration known	yes	yes	yes	no	yes
**Correct disinfectant concentration used**	no	no	no	no	yes
**Correct exposure time to cleaning agents**	no	no	no	yes	yes
Correct exposure time to disinfectants	yes	yes	yes	yes	yes
**Temperature of rinsing water controlled**	no	no	no	no	no
**Cleaning nozzle used appropriately**	yes	no	no	no	no
**Aerosol formation avoided**	yes	no	no	yes	no
**Work clothes changed between cleaning and disinfection procedures**	no	no	no	no	yes
Cleaning tools used exclusively during either cleaning or disinfection	no	yes	yes	no	yes
Disinfectant rotation reported in the manual	no	yes	yes	yes	yes
Different products used between cleaning and disinfection	no	yes	yes	no	yes
Appropriate rinsing of the surfaces used	yes	no	yes	yes	yes
Tools disassembled before cleaning	yes	no	yes	yes	yes
**Raw meat debris persistence avoided**	yes	no	no	no	no
**Dimension of drainage holes suitable for debris**	yes	no	no	no	yes
Product in use specified in the manual	yes	yes	yes	yes	yes
Operation performed according to the manual	yes	yes	yes	no	no
**All the surfaces of the plant cleaned**	no	no	yes	no	yes
Washability of all surfaces	yes	yes	yes	no	yes
**Drainage wells used appropriately**	yes	yes	no	no	no
Proper procedure reported in the manual	yes	yes	yes	yes	no

*C&D procedures disregarded by three or more plants are indicated in bold*.

### Isolation and identification of *Listeria monocytogenes*

A total of 169 environmental swabs were collected before and after the C&D procedures on 28 different surfaces. A number of 98 samples were gathered before and 71 after the C&D procedures. The number of samples collected in each plant varied according to the plant typology and to the number of the sampleable surfaces.

Overall 45 *L. monocytogenes* strains were isolated from the environmental swabs, 36 of them were retrieved before and nine after the C&D procedures.

In Table 2 all the sampled surfaces and the quantity of positive strains collected before or after the C&D procedures were reported.

**Table 2:** **Sampled surfaces and number of positive swabs among all of the collected swabs**.

**Sampled surfaces**	**Before C&D (n. positive**	**After C&D (n. positive**
	**swabs/total swabs)**	**swabs/total swabs)**
Bacon	0/1	0/0
Broom	0/0	1/1
Container of raw materials	1/4	0/1
Container of raw meat	1/2	0/1
Corner wall-floor	1/1	0/1
Drying room	1/3	0/0
Floor	6/8	0/8
Floor-drainage	1/2	1/2
Hook	0/1	0/0
Kart with meat	0/1	0/0
Kneader	3/8	1/5
Knife	3/10	0/3
Drainage of the refrigerating room	0/0	1/1
Drainage of the working room	1/1	1/1
Needle meat aerator	0/5	1/5
Meat mincer	0/10	0/6
Pallet with the bacon	0/1	0/0
Refrigerating room	1/5	0/2
Rope for hook mobility	0/0	0/1
Salami tying	2/5	0/4
Sausage maker	6/11	0/7
Sharpening steel	0/0	0/2
Sink	0/1	0/1
Spatula	0/1	0/0
Table	8/13	2/12
Under the table	0/1	0/2
Wall	0/2	0/4
Wall-Floor	0/0	1/1
Warehouse	1/1	0/0

The surfaces revealed to be contaminated with *L. monocytogenes* before the procedures were the following: corner wall-floor, refrigerating room, warehouse, knife, meat container, kneader, sausage maker, salami tying, drying room, floor, corner floor-drainage, drainage of the working room, container of raw materials.

The surfaces found positive to *L. monocytogenes* after the C&D were: meat aerator, kneader, corner wall-floor, corner floor-drainage, drainage of the refrigerating room, broom, table, drainage of the working room.

A subsample of 19 *L. monocytogenes* isolates, selected among the 45 positive samples were characterized. This subsample was selected to be representative of the surfaces where positivity for *L. monocytogenes* was found both before and after C&D (5 couple of isolates). Moreover, 4 and 5 strains isolated before and after C&D respectively, but being unrelated and representative of the whole variety of surfaces, were included.

### Antibiotics susceptibility testing

The entire selected subsample of *L. monocytogenes* was tested for its susceptibility to a panel of antibiotics. Results are described in Table 3. All investigated isolates resulted susceptible to the majority of the tested molecules.

**Table 3:** **Number of isolates categorized as sensitive, resistant, or intermediate toward a panel of antibiotics according to EUCAST cutoff**.

**Molecule**	**Sensitive**	**Resistant**	**Intermediate**
Ampicillin	19	0	0
Cefotaxime	11	2	6
Ceftriaxone	0	12	7
Daptomycin	19	0	0
Erythromycin	19	0	0
Levofloxacin	19	0	0
Linezolid	19	0	0
Meropenem	19	0	0
Moxifloxacin	19	0	0
Penicillin	19	0	0
Teicoplanin	19	0	0
Vancomycin	19	0	0

### Genotypic assay

The assessed genetic resistance determinants to biocide and heavy metals are reported in Table 4. Results showed the panpresence of *lde* in the entire tested sample of strains and a widespread presence of *mdrL* and *cadA1-Tn5422* found in 15 and 11 out of 19 *L. monocytogenes* strains respectively. Less than half of the sample harbored *F2365_2257* (7/19) and three strains *LMOSA_*2330. A total of 8 strains displayed the presence of three resistance determinants (*lde, mdrL* and *cadA1*-*Tn5422*), being the most frequent genotype among the investigated sub-sample (Table 4).

**Table 4:** **Presence of the tested genetic determinants of resistance among the selected strains**.

**Gene**	**Present**	**Not present**
*mdrL*	15	4
*lde*	19	0
*cadA1-Tn5422*	11	8
*cadA2-pLM80*	0	19
*cadA3-EGDe*	0	19
*LMOSA_2330*	3	16
*LMOSA_2220*	0	19
*pLI37*	0	19
*F2365_2257*	7	12

### Typing of the *Listeria monocytogenes* isolates

Table 5 reported the number of strains of each Sequence Type, according to MLST protocol. The obtained sequence types were ST 1 (1/19), ST 9 (12/19), ST 26 (1/19), ST 121 (3/19), ST 489 (1/19), ST 517 (1/19). Among the five couple of isolates found, both before and after C&D, only two couples displayed the same sequence type (ST9). The rest belonged to different sequence types suggesting their unrelatedness. Regarding the isolated tested for their representativeness of the sampled surfaces, the most widespread type was ST9, found in six samples, of which four belonging to the post C&D isolates.

**Table 5:** **Number of strains for each sequence type among the selected strains**.

**Sequence Type**	**Number of Strains**
1	1
9	12
26	1
121	3
489	1
517	1

**Figure 1:**
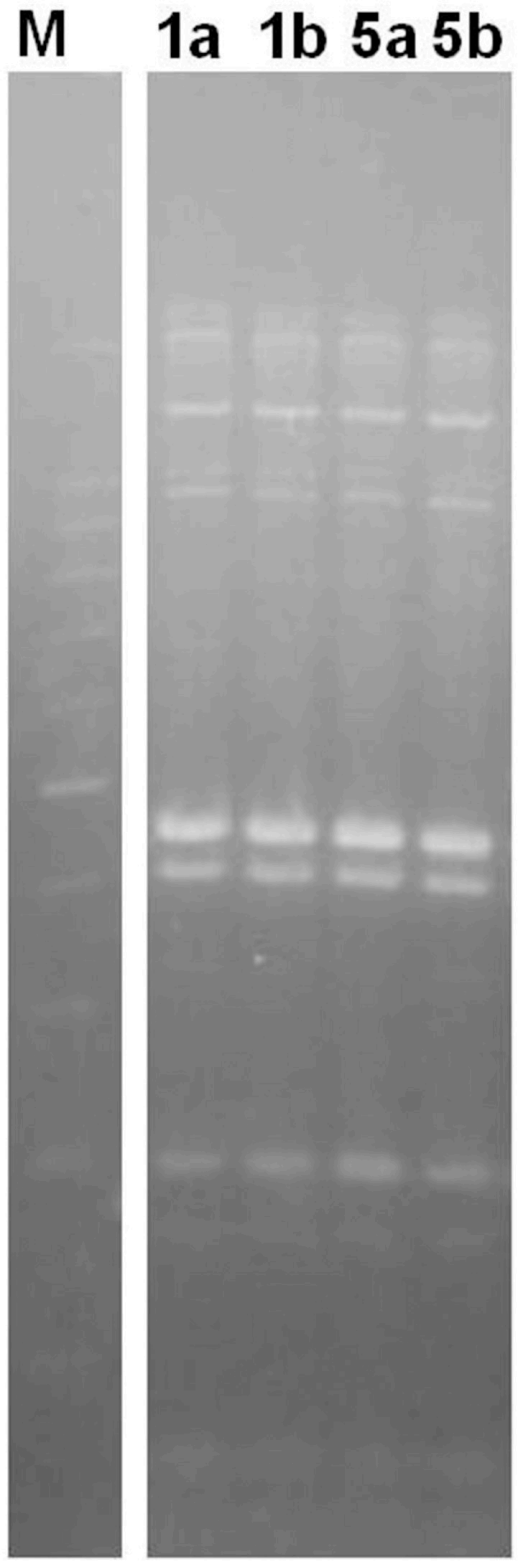
**ERIC profile of the selected strains**. M: 100 bp DNA Ladder H3 RTU (Nippon Genetics, Germany); Numbers indicate the tested strains.

In order to obtain a deeper insight into the differences among the two couples of strains (1a/1b and 5a/5b) found to be related in terms of sequence type, their ERIC profile was investigated. The results showed a complete overlap in the ERIC profiles of the two couples of tested strains (Figure 1) thus suggesting the potential for their identity. One couple (1a/1b) was selected for the gene expression assay.

### Gene expression profiling

**Figure 2:**
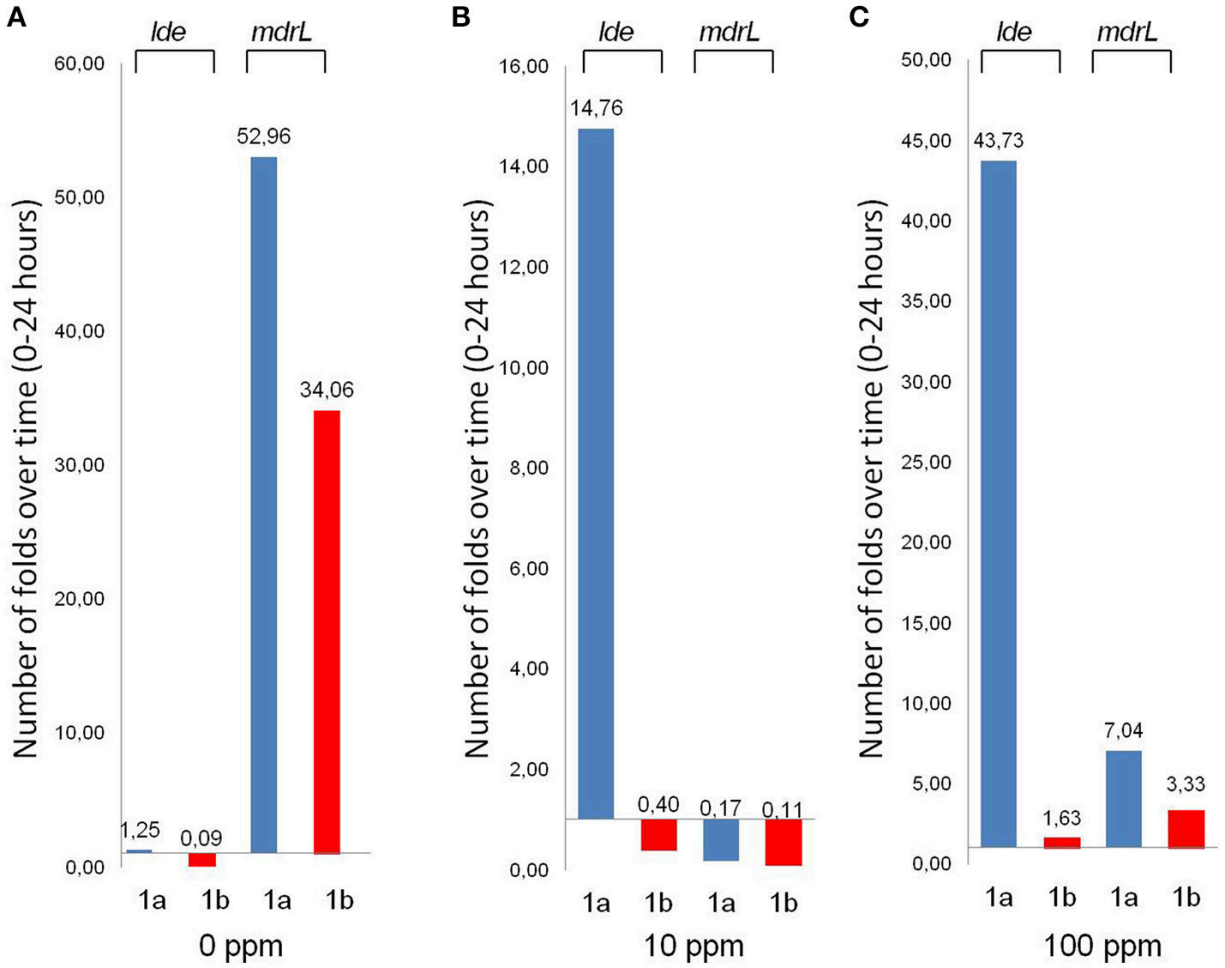
**Gene expression profile of strains 1a (isolated before C&D procedures) and 1b (isolated after C&D procedures), at concentration levels of 0 ppm (A), 10 ppm (B), 100 ppm (C) of Benzalkonium Chloride (BC)**. Histograms represent the number of folds *lde* and *mdrL* expressed are over time, taking into account the difference between time point 24 h and time point 0.

The *lde* and *mdrL* activities of two strains, 1a and 1b, collected before and after C&D procedures from the same table, were further studied using gene expression profiling. Genotypic and phenotypic characteristics of the investigated strains were reported in Table S2. Gene expression was evaluated calculating the increase or decrease in expression over time of the two target genes in the absence (control) and presence (cases) of benzalkonium chloride (BC) treatment (10 and 100 ppm). The results were normalized against the expression of *tufA* gene (housekeeping) both in the treatment and control groups. As can be noticed from Figure 2, the two tested strains, 1a and 1b, displayed different expression profiles for the two investigated genes.

*Lde* was expressed mainly by 1a strain (isolated before the C&D procedures), in the presence of BC exposure, with a dose-dependent trend. On the contrary, strain 1b (isolated after the C&D procedures) displayed a low and constant trend of expression with a slight increase in the presence of the maximum BC tested dose (100 ppm; Figures 2A–C). In the case of *mdrL*, a different scenario was observed. The two strains presented a similar expression profile with the maximum expression displayed in the absence of BC and a strong reduction of the gene expression showed under exposure to BC at 10 and 100 ppm (Figures 2A–C). A slight increase in the gene expression was observed in the presence of the maximum dose of BC, if compared with the medium used concentration (Figures 2B,C).

Results were described in Figure S1 and Figure 2.

## Discussion

The results presented in this work highlight the importance of the implementation of correct C&D procedure to reduce the potential of selection of bacterial resistance to biocides.

The hygiene assessment of the five selected plants emphasized many critical actions disregarded during C&D procedures. Among these were the (i) wrong concentration of disinfectant, (ii) the incorrect exposure time to the cleaning agents, (iii) the absence of systems to control the temperature of rinsing water and (iv) the presence of physical obstacles to debris and wastewater elimination resulted the most common, as well as the most prone to boost the development of bacterial persistence in the environment (Muhterem-Uyar et al., 2015). The consequent finding of raw meat debris on working surfaces, observed in more than half of the studies cases, depicted a scenario where raw leftover meat acting as bacterial reservoir improved the formation of ecological niches where C&D agents did not penetrate (Thévenot et al., 2006). In addition to this, the improper use of high-pressure hoses, observed in the majority of the plants, provoked the spreading of aerosol particles belonging to drainage water, thus representing a possible source of environmental bacterial contamination.

These observations could strongly justify the high number of *L. monocytogenes* strains isolated after the C&D procedures (Muhterem-Uyar et al., 2015).

A high variety of STs was found in the typed subsample of *L. monocytogenes*. This is coherent with the heterogeneity of *L. monocytogenes* strains observed in other studies (Parisi et al., 2010).

Genotyping analysis displayed the widespread presence of *lde* and *mdrL* in the tested isolates (19/19 and 15/19 respectively) thus suggesting a potential for their tolerance to biocidal compounds.

On the contrary the reduced occurrence of heavy metals resistance genes such as *cadA1* (11/19), *F2365_2257* (7/19) and *LMOSA_2330* (3/19) could be related to a low presence of heavy metals in the plants environment (Mullapudi et al., 2008).

The same ST was shared by two couples of strains collected after and before the C&D procedures. These strains were probably resident in the environment before the C&D procedures and, due to the low effectiveness of such procedures, recolonized the recently washed surfaces. These strains were also found identical according to the ERIC profile, thus highlighting the possibility for an authentic similarity from a genetic point of view.

The analysis of the gene expression profiles in the presence of BC suggested the possibility for a differential tolerance to the two tested strains. Despite the genetic similarity of the two strains, the impairment in their expression of lde gene was observed, thus suggesting a lack of phenotypic relatedness between the two strains.

The strain isolated before the C&D procedures displayed an *lde* expression linearly related to the entity of the exposure, suggesting the possibility for a protective effect of the gene product over time. In the case of the strain isolated after the C&D procedures, *lde* gene was expressed only in the presence of high concentration of BC, suggesting an improved tolerance of the strain to the biocide. This is in line with the results of other authors that reported a different expression rate between strains but with a lower extent in the expression of the targeted genes (Romanova et al., 2006; Tamburro et al., 2015). Nevertheless, Tamburro et al. found high transcription levels of both *mdrL* and *lde* efflux systems in selected BC-resistant strains of *Listeria monocytogenes*, where the *lde* gene presented the highest expression level among the tested strains (Tamburro et al., 2015). However, both in the work of Romanova et al, and Tamburro et al., the gene expression profiles were evaluated after few minutes of exposure to BC.

The similar *mdrL* expression profile displayed by the two tested strains, being maximum in the absence and inhibited in the presence of BC, suggested a role for this gene different from the tolerance enhancement under biocide stress.

Overall the results of this study suggest a possible role for C&D procedures to select *L. monocytogenes* persisters pointing out the importance of dealing with the identification of risk factors in the food plants sanification procedures that might select more tolerant strains. Bacterial tolerance to biocides through the activation of efflux-mediated mechanism of protection is becoming an emerging threat in the food chain as also reported by other authors (Ortega Morente et al., 2013) It has, however, yet to be discovered how many efflux pump families could be involved in this phenomena and how they can complement. Further studies are necessary to unravel this point.

## Author contributions

DC and CL conceived the experiments, conducted the experiments, analyzed the results and wrote the manuscript, AR and VC conceived the experiments, analyzed the results and wrote the manuscript, EC and AD conducted the experiments, GC and VG conceived the experiments and analyzed the results. All authors reviewed the manuscript.

### Conflict of interest statement

The authors declare that the research was conducted in the absence of any commercial or financial relationships that could be construed as a potential conflict of interest.
