# Supplementary material for: Resistance to Biocides in Listeria monocytogenes Collected in Meat-Processing Environments
Source: Front Microbiol. 2016 Oct 19;7:1627. doi: 10.3389/fmicb.2016.01627 (PMC5069283; doi:10.3389/fmicb.2016.01627)
Supplement: Supplementary file 1 [file Table1.pdf]

| Target name | Primer sequence (5'-3') | Annealing temperature (°C) | Reference |
|---|---|---|---|
| cadA1-Tn5422 | 1- CAGAGCATTTACTGACCATCAATCGTT<br>2- TCTTCTTCATTTAACGTTCCAGCAAAAA | 55 | Mullapudi, S., Siletzky, R. M., & Kathariou, S. (2010). Diverse cadmium resistance determinants in Listeria monocytogenes isolates from the turkey processing plant environment. *Applied and Environmental Microbiology*, *76*(2), 627–30. http://doi.org/10.1128/AEM.01751-09 |
| cadA2-pLM80 | 1- ACAAGTTAGATCAAAAGAGTCTTTTATT<br>2- ATCTTCTTCATTTAGTGTTCCTGCAAAT | 55 | Mullapudi, S., Siletzky, R. M., & Kathariou, S. (2010). Diverse cadmium resistance determinants in Listeria monocytogenes isolates from the turkey processing plant environment. *Applied and Environmental Microbiology*, *76*(2), 627–30. http://doi.org/10.1128/AEM.01751-09 |
| cadA3-EGDe | 1- TGGTAATTTCTTTAAGTCATCTCCCATT<br>2- GCGATGATTGATAATGTCGATTACAAAT | 55 | Mullapudi, S., Siletzky, R. M., & Kathariou, S. (2010). Diverse cadmium resistance determinants in Listeria monocytogenes isolates from the turkey processing plant environment. *Applied and Environmental Microbiology*, *76*(2), 627–30. http://doi.org/10.1128/AEM.01751-09 |
| LMOSA_2330 | 1- GCATACGTACGAACCAGAAG<br>2- CAGTGTTTCTGCTTTTGCTCC | 55 | Lee, S., Rakic-Martinez, M., Graves, L. M., Ward, T. J., Siletzky, R. M., & Kathariou, S. (2013). Genetic determinants for cadmium and arsenic resistance among Listeria monocytogenes serotype 4b isolates from sporadic human listeriosis patients. *Applied and Environmental Microbiology*, *79*(7), 2471–6. http://doi.org/10.1128/AEM.03551-12 |
| LMOSA_2220 | 1- CAACTTTGACCCTGTGGAG<br>2- CTTTCCATTCAATCACTGCG | 55 | Lee, S., Rakic-Martinez, M., Graves, L. M., Ward, T. J., Siletzky, R. M., & Kathariou, S. (2013). Genetic determinants for cadmium and arsenic resistance among Listeria monocytogenes serotype 4b isolates from sporadic human listeriosis patients. *Applied and Environmental Microbiology*, *79*(7), 2471–6. http://doi.org/10.1128/AEM.03551-12 |
| pLI37 | 1- CAACCAGATCAGTTACCATTAAC<br>2- TGCTTCTCCAGAGATTTCTTCTG | 55 | Lee, S., Rakic-Martinez, M., Graves, L. M., Ward, T. J., Siletzky, R. M., & Kathariou, S. (2013). Genetic determinants for cadmium and arsenic resistance among Listeria monocytogenes serotype 4b isolates from sporadic human listeriosis patients. *Applied and Environmental Microbiology*, *79*(7), 2471–6. http://doi.org/10.1128/AEM.03551-12 |
| F2365_2257 | 1- ACATTGCGAGAACACCTTGG<br>2- GATTTATCGGCGCAATGACG | 55 | Lee, S., Rakic-Martinez, M., Graves, L. M., Ward, T. J., Siletzky, R. M., & Kathariou, S. (2013). Genetic determinants for cadmium and arsenic resistance among Listeria monocytogenes serotype 4b isolates from sporadic human listeriosis patients. *Applied and Environmental Microbiology*, *79*(7), 2471–6. http://doi.org/10.1128/AEM.03551-12 |
| mdrL | 1- TTTCGAGCTGGTTGGG<br>2- CACTAACGCGTGTGATACTTT | 50 | Romanova, N. A., Wolffs, P. F. G., Brovko, L. Y., & Griffiths, M. W. (2006). Role of Efflux Pumps in Adaptation and Resistance of Listeria monocytogenes to Benzalkonium Chloride. Applied and Environmental Microbiology, 72(5), 3498–3503. http://doi.org/10.1128/AEM.72.5.3498 |
| lde | 1- ATCCTCATATAACTCAAGCG<br>2- CAATGGCTTTCGCACAA | 50 | Romanova, N. A., Wolffs, P. F. G., Brovko, L. Y., & Griffiths, M. W. (2006). Role of Efflux Pumps in Adaptation and Resistance of Listeria monocytogenes to Benzalkonium Chloride. Applied and Environmental Microbiology, 72(5), 3498–3503. http://doi.org/10.1128/AEM.72.5.3498 |
| tufA | 1- GCTGAAGCTGGCGACAACA<br>2- CTTGACCACGTTGGATATCTTCAC | 58 | Tamburro, M., Ripabelli, G., Vitullo, M., Dallman, T. J., Pontello, M., Amar, C. F. L., et al. (2015). Gene expression in Listeria monocytogenes exposed to sublethal concentration of benzalkonium chloride. *Comp. Immunol. Microbiol. Infect. Dis.* 40, 31–39. doi:10.1016/j.cimid.2015.03.004. |

**Table S1.** Primer, temperature of annealing and references for each investigated gene.
