# Supplementary material for: Resistance to Biocides in Listeria monocytogenes Collected in Meat-Processing Environments
Source: Front Microbiol. 2016 Oct 19;7:1627. doi: 10.3389/fmicb.2016.01627 (PMC5069283; doi:10.3389/fmicb.2016.01627)
Supplement: Supplementary file 2 [file Table2.pdf]

Strain	1a	1b
Ampicillin	S	S
Cefotaxime	S	S
Ceftriaxone	I	I
Daptomycin	S	S
Erythromycin	S	S
Levofloxacin	S	S
Linezolid	S	S
Meropenem	S	S
Moxifloxacin	S	S
Penicillin	S	S
Teicoplanin	S	S
Vancomycin	S	S
MdrL	+	+
Lde	+	+
cadA1-Tn5422	+	+
cadA2-pLM80	-	-
cadA3-EGDe	-	-
LMOSA_2330	-	-
LMOSA_2220	-	-
pLI37	-	-
F2365_2257	-	-
Sequence Type	9	9

Table S2. Genotypic and phenotypic characteristics of strains 1a and 1b.
