# Supplementary material for: Resistance to Biocides in Listeria monocytogenes Collected in Meat-Processing Environments
Source: Front Microbiol. 2016 Oct 19;7:1627. doi: 10.3389/fmicb.2016.01627 (PMC5069283; doi:10.3389/fmicb.2016.01627)
Supplement: Supplementary file 3 [file Image1.PDF]

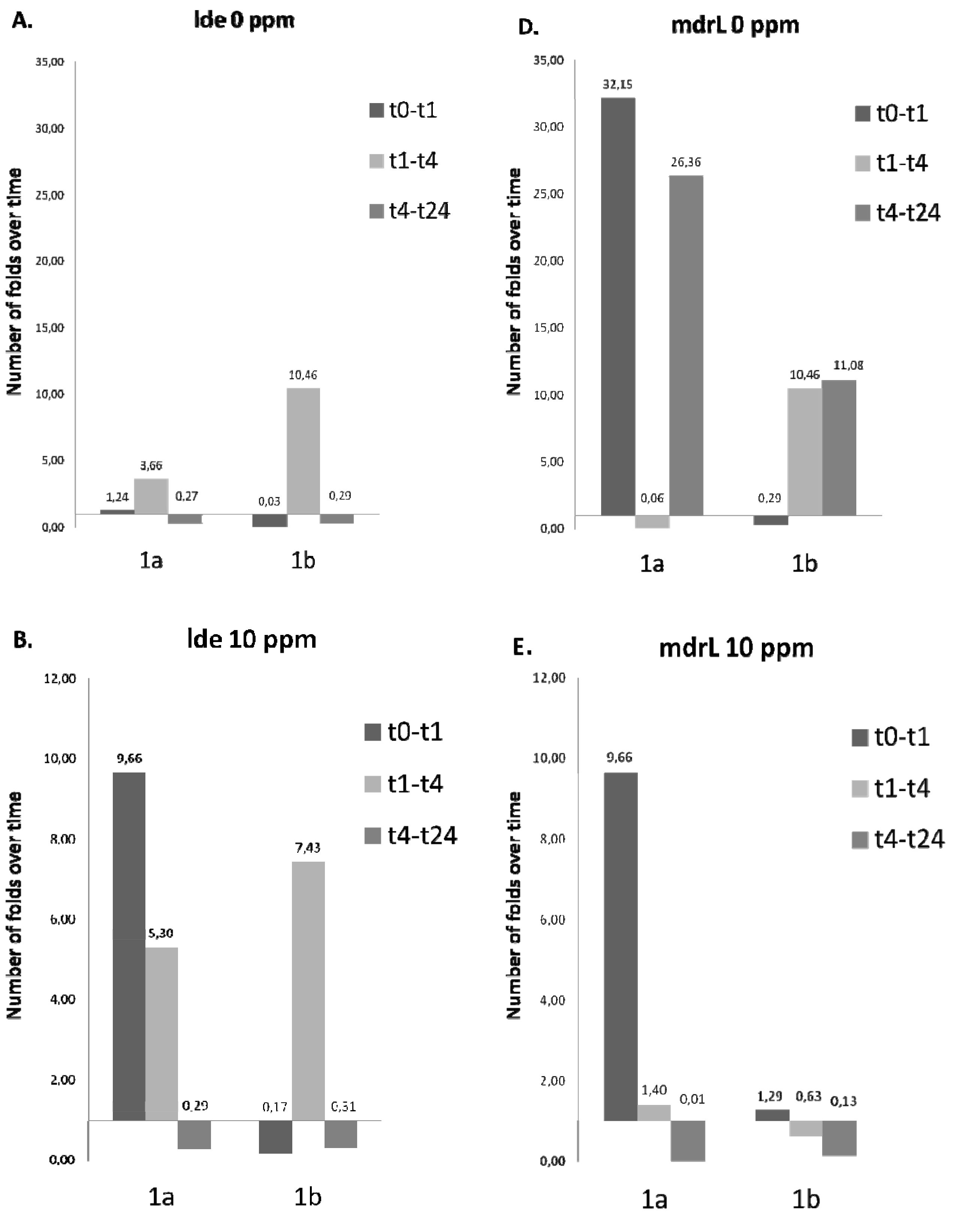
A.
lde 0 ppm
D.
mdrL 0 ppm
B.
lde 10 ppm
E.
mdrL 10 ppm
Number of folds over time
t0-t1
t1-t4
t4-t24
1a
1b
1,24
3,66
0,27
0,03
10,46
0,29
32,15
0,06
26,36
0,29
10,46
11,08
9,66
5,30
0,29
0,17
7,43
0,31
9,66
1,40
0,01
1,29
0,63
0,13
35,00
30,00
25,00
20,00
15,00
10,00
5,00
0,00
12,00
10,00
8,00
6,00
4,00
2,00
0,00

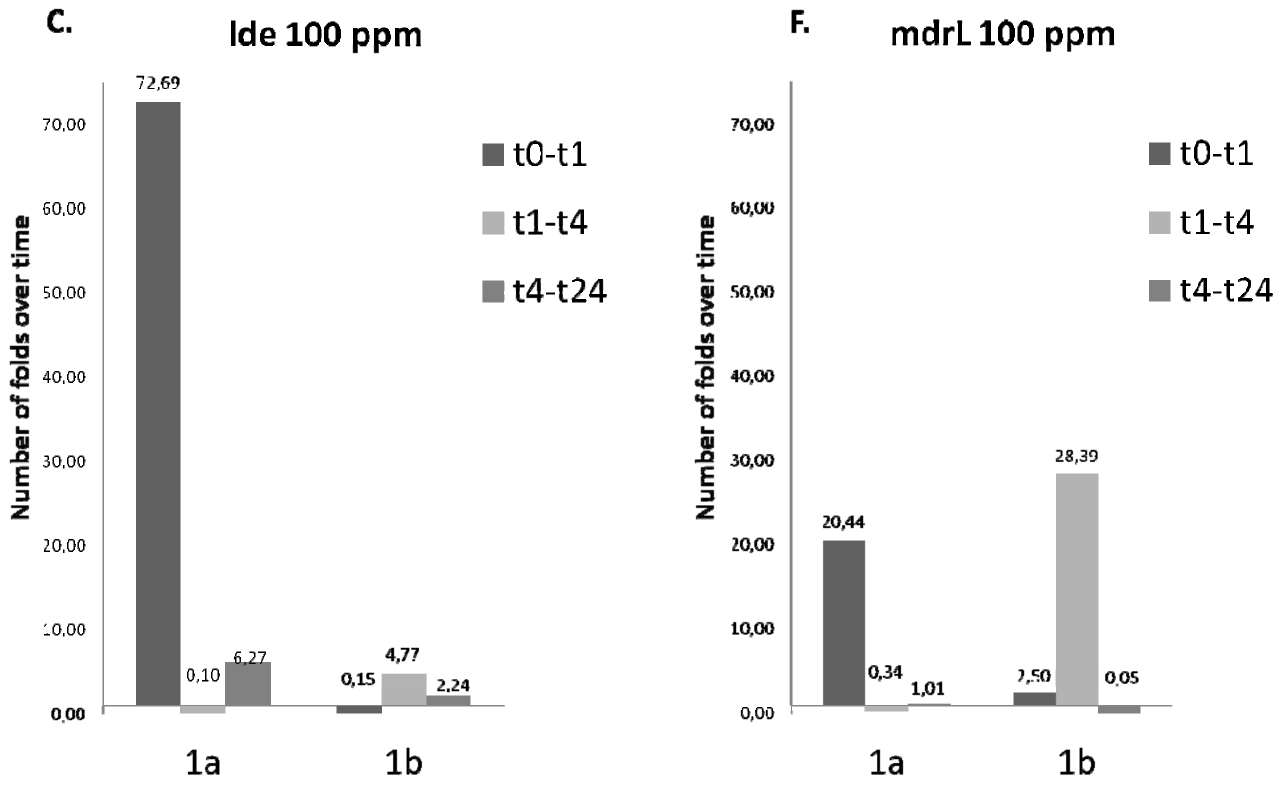
C.
lde 100 ppm
72,69
70,00
60,00
50,00
40,00
30,00
20,00
10,00
0,00
Number of folds over time
0,10
6,27
0,15
4,77
2,24
1a
1b
t0-t1
t1-t4
t4-t24
F.
mdrL 100 ppm
20,44
0,34
1,01
2,50
28,39
0,05

Figure 1S: Gene expression profile over time

In the absence of BC (0 ppm) the maximum increment in the expression of *lde* gene was noticed after 4 hours (t4), if compared with the preceding time point (t0), in both the tested strains (pre and post C&D procedures) (Figure 1S, panel A). However the trend of *lde* expression appears to be different in the two strains. The strain isolated during the pre- C&D procedures (1a) showed an increasing trend in the first phases of the experiment (t1-t0, t4-t1 hours) and then a decreasing in the last phase (t24-t4), on the contrary, the strain 1b, isolated after C&D procedures, displayed a decreasing trend even in the first phase of the experiment (t1-t0). The same pattern was displayed by strain 1b, even when exposed to BC at a concentration of 10ppm. In the same condition strain 1a showed a strong increasing trend all over the time points with a slight decreasing after 24 hours of incubation (t24-t4) (Figure 1S, panel B). Finally, in the presence of BC at the highest tested concentration (100 ppm), strain 1a displayed an increasing trend in the first phase of the time course (t1-t0), a slight reduction at the time point t4-t1 and then a slight increasing at the end of the exposure time (t24-t4) (Figure 1S, panel C). On the contrary, strain 1b exerted an initial reduction in the trend of expression (t1-t0) and a increasing trend of expression at the remaining time points (t4-t1, t24-t4).

Concerning the expression of *mdrL* gene, the two tested strains exert a different extent in the expression over time and depending on BC concentration. In the absence of BC, an increasing trend of expression was shown by both 1a and 1b strains but with a difference in the timing of gene expression the increased of *mdrL* expression was displayed only at t1 and t24 in the case of strain 1a and for t4 and t24, in the case of strain 1b (Figure 1S, panel D). In the presence of BC 10 ppm a decreasing trend was evinced by both the tested strains even if with a different extent between them (Figure 1S, panel E). Finally, in the presence of 100 ppm BC, a different trend was noticed between the 1a and 1b strains with the first one being increasing only at t1 and the second one showing an increasing trend in the first phase of the exposure (t1-t0, t4-t1) (Figure 1S, panel F).
